# Nano-Photothermal ablation effect of Hydrophilic and Hydrophobic Functionalized Gold Nanorods on *Staphylococcus aureus* and *Propionibacterium acnes*

**DOI:** 10.1038/s41598-018-24837-7

**Published:** 2018-05-02

**Authors:** Nouf N. Mahmoud, Alaaldin M. Alkilany, Enam A. Khalil, Amal G. Al-Bakri

**Affiliations:** 1 Department of Pharmaceutics & Pharmaceutical Technology, School of Pharmacy, The University of Jordan, Amman, 11942 Jordan; 2 Department of Pharmacy, Faculty of Pharmacy, Al-Zaytoonah University of Jordan, Amman, 11733 Jordan

## Abstract

The potential photothermal bactericidal activity of hydrophilic functionalized poly ethylene glycol (PEG)-gold nanorods (GNR) and hydrophobic functionalized polystyrene (PS)-GNR was evaluated towards strains of *Staphylococcus aureus* (*S*. *aureus*) and *Propionibacterium acnes* (*P*. *acnes*) by measuring the percentage reduction of bacterial viable count upon GNR excitation with a near infra-red (NIR) laser beam. Our results suggest that functionalized GNR had a minimal bactericidal activity against *S*. *aureus* and *P*. *acnes* (≤85%, i.e. ≤1 log_10_ cycle reduction of bacterial viable count). However, the local heat generated upon exciting the functionalized GNR with NIR laser beam has a significant photothermal ablation effect (≥99.99%, i.e. ≥4 log_10_ cycles reduction of bacterial viable count). Such photothermolysis effect could potentiate the antibacterial activity of GNR, which may call for minimum concentration and side effects of these nanotherapeutics.

## Introduction

Several nanotechnology-based modalities have made important contributions to biomedical and nanomedicine fields^1^. For example, different therapeutic nanoparticle platforms, such as liposomes, albumin nanoparticles and polymeric micelles, have been approved for cancer treatment^2–5^. Others including organic and inorganic nanoparticles and DNA-based nanoparticles were investigated for drug delivery, sensing, labelling and tracking in biological systems, in addition to diagnosis and treatment of cancers and bacterial infections^6–11^.

Photothermal therapy is considered as the central application of plasmonic nanoparticles in medicine. Plasmonic nanoparticles absorb light efficiently in the near infra-red (NIR) region, where light penetration in tissues is optimal, and convert it to local heat by photo-exciting the conduction electrons to induce surface plasmon oscillations followed by non-radiative relaxation^12–14^. Among the plasmonic nanomaterials, gold nanoparticles (GNP) have received considerable attention in photothermal therapy due to their high photothermal conversion efficiency, low toxicity, easy synthesis and functionalization and easily tuned surface plasmon resonance frequency to NIR region^15,16^.

Among all plasmonic GNP, the gold nanorods (GNR), exhibit the most ideal NIR absorption cross section and demonstrate extremely efficient NIR photothermal heat conversion^17,18^. The most common size of GNR utilized for use in photothermal is around 40 nm in length and 10 nm in diameter, with a longitudinal plasmon resonance around 800 nm. The increase in the local temperature around the particles and the targeted cells is depending on the laser power, time of exposure, and concentration of GNP^19^. Photothermally induced cell death can take place via apoptosis or necrosis that results from denaturation or breakdown of proteins, cell cavitation, cellular structure rupturing, evaporation of cellular liquid and bubble formation by shock waves^20–22^.

It is known that more than 70% of bacterial infections are resistant to conventional antibiotics^23^. With this in mind, development of new and effective antimicrobial agents is an urgent need. The resulted local hyperthermia of GNR could be efficient in eliminating microorganisms selectively by combining the use of laser beam and functional GNR by disruption the bacterial cell membrane, fatty acid melting, and protein denaturation^24^.

The photothermal-based ablation activity of gold nanoparticles towards skin pathogens such as *Staphylococcus aureus* (*S*. *aureus*) was explored previously, however, those addressing other skin pathogens such as *Propionibacterium acnes* (*P*. *acnes*) were lacking in the literature. For example, the selective *in vitro* photothermal nano-therapy of *S*. *aureus* or methicillin-resistant *S*. *aureus* (MRSA) infections mediated by antibodies conjugated gold nanoparticles was investigated in previous studies^19,25–27^. Recently, the photothermal lysis of pathogenic bacteria such as *S*. *aureus* by platinum nanodots decorated GNR was investigated^28^.

Moreover, GNP demonstrated an intrinsic antimicrobial activity, the degree and the spectrum of which are dependent on their surface chemistry. For example, cationic and hydrophobic functionalized GNP effectively suppressed growth of 11 clinical multi drug resistant isolates, including both Gram-negative and Gram-positive bacteria^29^. Although the antibacterial activity of GNP of different surface chemistry was investigated in many previous reports^30,31^, those addressing the antibacterial activity of hydrophobic GNP were rare in the literature.

In the current work, we demonstrated the effectiveness of the hyperthermia of GNR in killing *S*. *aureus* and *P*. *acnes* as the most common bacteria responsible for the pathogenesis of acne vulgaris. In light of our previous findings, where we successfully targeted the skin hair follicles with both hydrophilic and hydrophobic GNR^32^, PEG-GNR and PS-GNR could be promising candidates to treat hair follicle diseases such as acne vulgaris. Besides, the bactericidal activity of the hydrophobic GNR (PS-GNR) was evaluated by employing a simple and novel design without the need to use solubilizing agents or surfactants that may complicate the measurements and increase the artifacts.

## Results and Discussion

### Characterization of CTAB-GNR, PEG-GNR and PS-GNR

**Figure 1:**
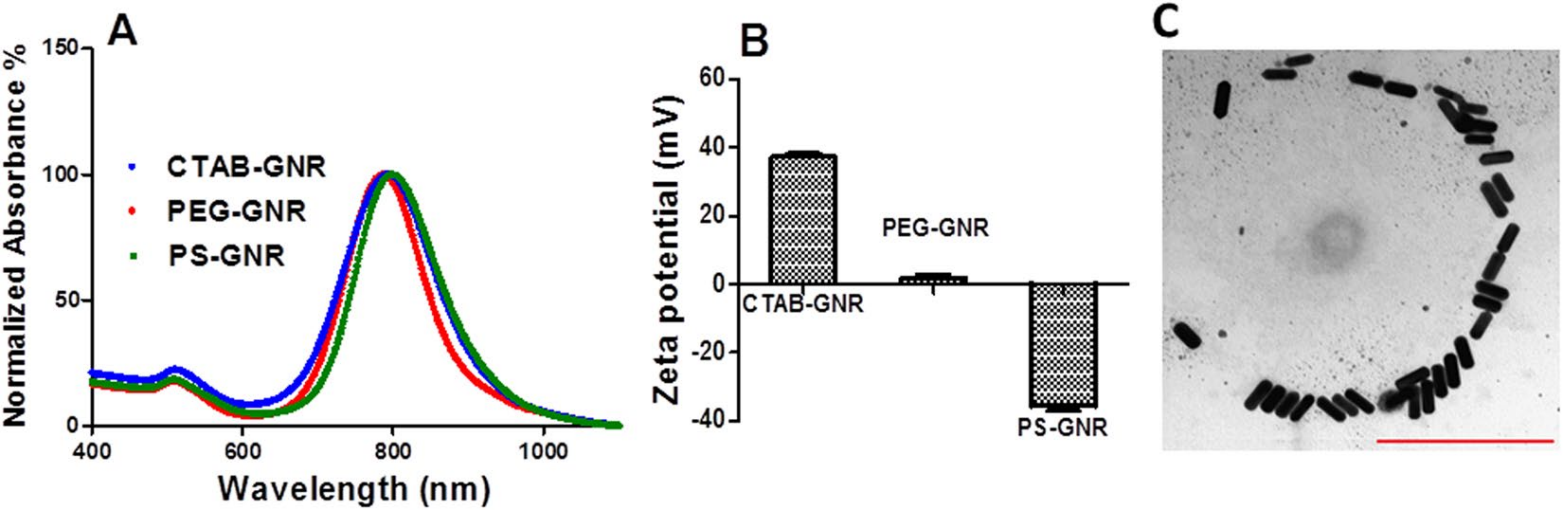
Characterization of GNR of different surface chemistry as labelled. (**A**) UV-vis absorption spectra of GNR suspensions. (**B**) Effective surface charge of GNR functionalized with different ligands. (**C**) TEM image of PEG-GNR features lengths of 38.2 ± 1.8 nm and widths of 10.1 ± 0.8 nm. Scale bar = 500 nm.

CTAB-GNR were successfully synthesized using seed mediated method and functionalized into hydrophilic PEG-GNR and hydrophobic PS-GNR. The prepared GNR solutions were characterized by UV-vis absorption spectroscopy, zeta potential, and transmission electron microscope (TEM). The UV-vis absorption spectra demonstrated typical transverse and longitudinal plasmon peaks of CTAB-GNR (510 nm and 792 nm, respectively) (Fig. 1A). Upon surface functionalization with PEG or PS, no significant broadening or tailing of the longitudinal peaks was observed, indicating the stability of the functionalized GNR suspensions. The slight red shift in the plasmon longitudinal peak observed for PS-GNR (800 nm) was related to the change in the refractive index of the suspending solvent from water to toluene.

In addition, the effective surface charges of the functionalized GNR confirmed successful functionalization. CTAB-GNR demonstrated a positive surface charge (+37 mV), whereas PEG-GNR featured an almost neutral surface charge (+1.7 mV) (Fig. 1B). The zeta potential of PS-GNR was measured in toluene and it was −35.6 mV (Fig. 1B). The shape and size of the functionalized GNR were confirmed by TEM. A TEM image of PEG-GNR featured lengths of ~38 nm and widths of ~10 nm, corresponding to an aspect ratio of ~3.8 (Fig. 1C).

### The configuration and setup of the experiment

In the current study, the photothermal-based antibacterial activity of two GNR suspensions; hydrophilic PEGylated and hydrophobic PS functionalized GNR was evaluated.

**Figure 2:**
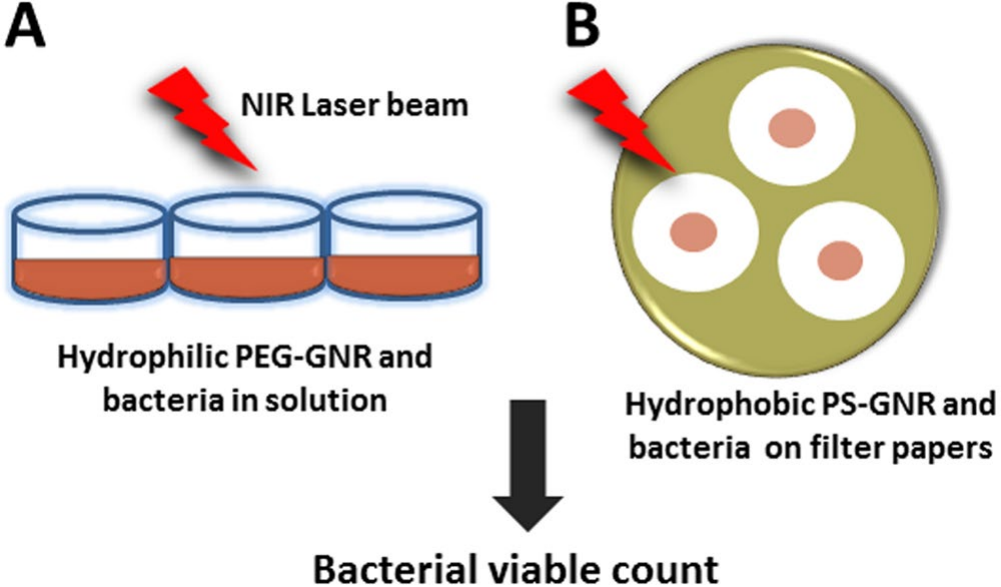
Cartoon demonstrating the different methods employed to evaluate the bactericidal activity of functionalized GNR; (**A**) Hydrophilic PEG-GNR were mixed with bacterial suspension in 96 well plates, while (**B**) Hydrophobic PS-GNR were exposed to the tested bacteria directly using sterile cellulose filter membranes.

We have previously reported the antibacterial activity of GNR coated with different hydrophilic ligands^33^. However, the antibacterial activity of hydrophobic GNR was rarely evaluated in the literature. In this study, we employed an optimized and simple method to investigate the antibacterial activity of the hydrophobic PS-GNR, since the commonly applied methods using different concentrations of dimethyl sulfoxide or surfactants (such as Tween 80) failed to improve the suspendability of PS-GNR within the bacterial suspension^34,35^. In this method, sterile cellulose filter membranes were used as matrices that allow a reasonable contact between hydrophobic PS-GNR and bacteria and support the bacterial growth by nutrient diffusion **(**Fig. 2**)**.

**Figure 3:**
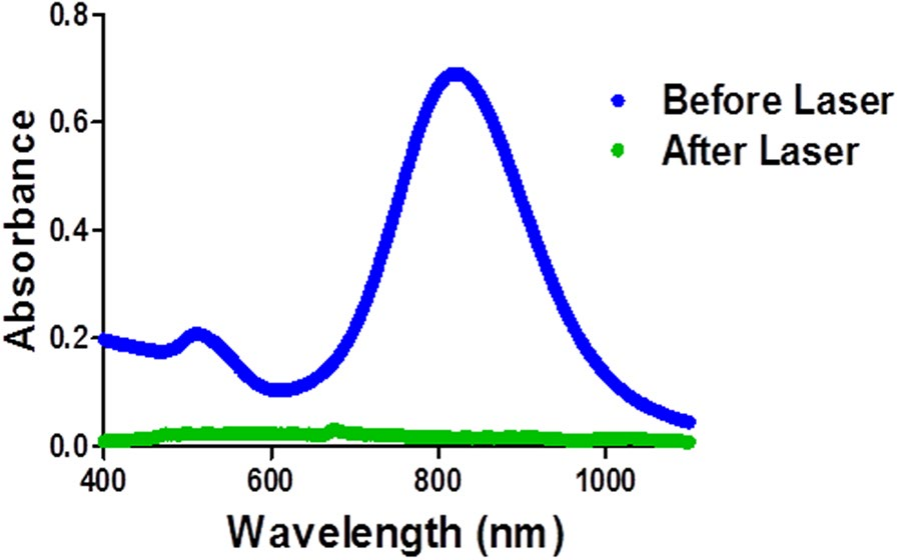
UV-vis absorption spectra of PEG-GNR before laser exposure (blue line) and after 20 min of laser exposure (green line) where a complete loss of the typical optical properties of GNR was observed.

The bactericidal activity of the functionalized GNR alone or upon exposure to laser was expressed as a percentage reduction of bacterial viable count. The time of NIR laser exposure was optimized to 15 min, since the exposure time of less than 5 min resulted in no significant difference of bacterial viability, while the exposure of more than 15 min resulted in a complete loss of the characteristic optical properties of GNR. Figure 3 shows the absence of typical UV-vis plasmon peaks of PEG-GNR after 20 min of laser exposure compared to UV-vis plasmon peaks of PEG-GNR prior to laser exposure.

### The bactericidal activity of PEG-GNR and PS-GNR alone or in combination with laser beam on *S*. *aureus* and *P*. *acnes*

**Figure 4:**
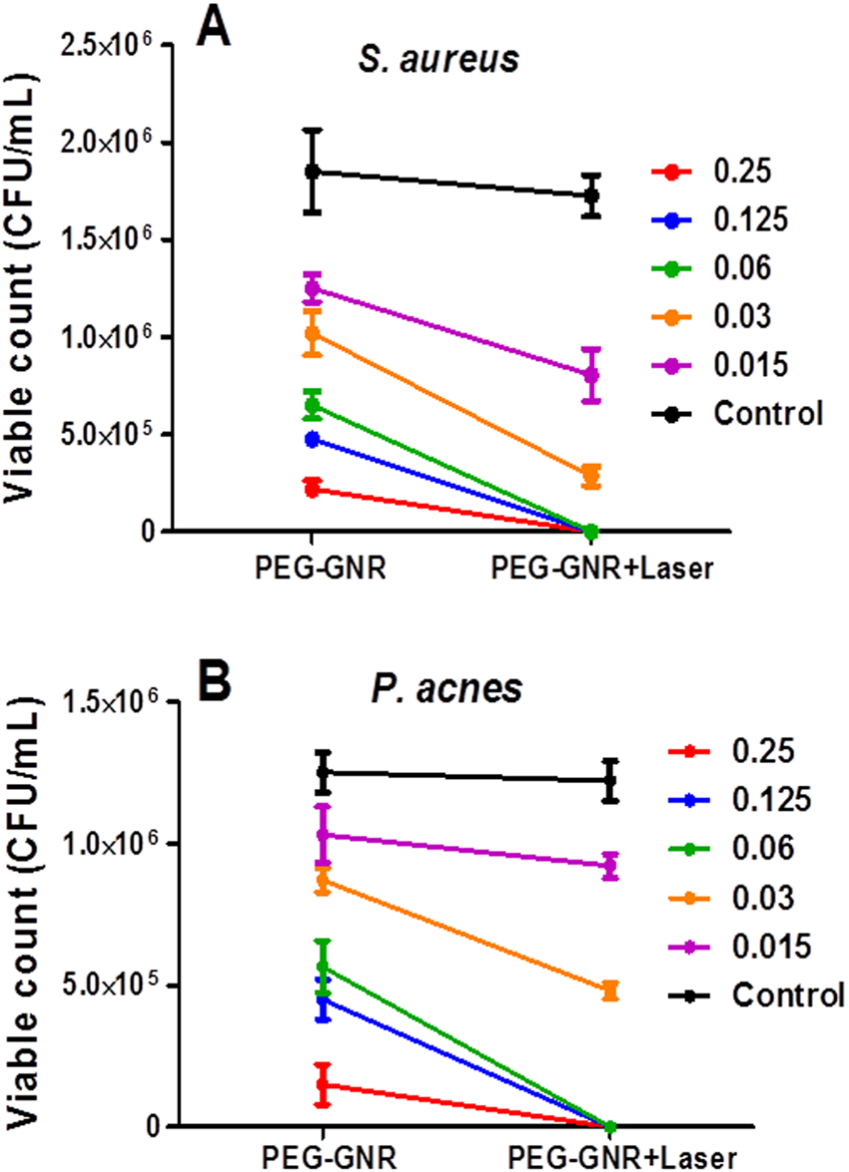
Effect of NIR laser exposure of bacteria pre-treated with PEG-GNR. (**A)** Bacterial viable count of *S*. *aureus* treated with different concentrations (nM) of PEG-GNR compared to those treated with PEG-GNR followed by exposure to laser beam. (**B**) Bacterial viable count of *P*. *acnes* treated with different concentrations (nM) of PEG-GNR compared to those treated with PEG-GNR followed by exposure to laser beam. Control: bacteria exposed to laser beam alone without GNR.

**Figure 5:**
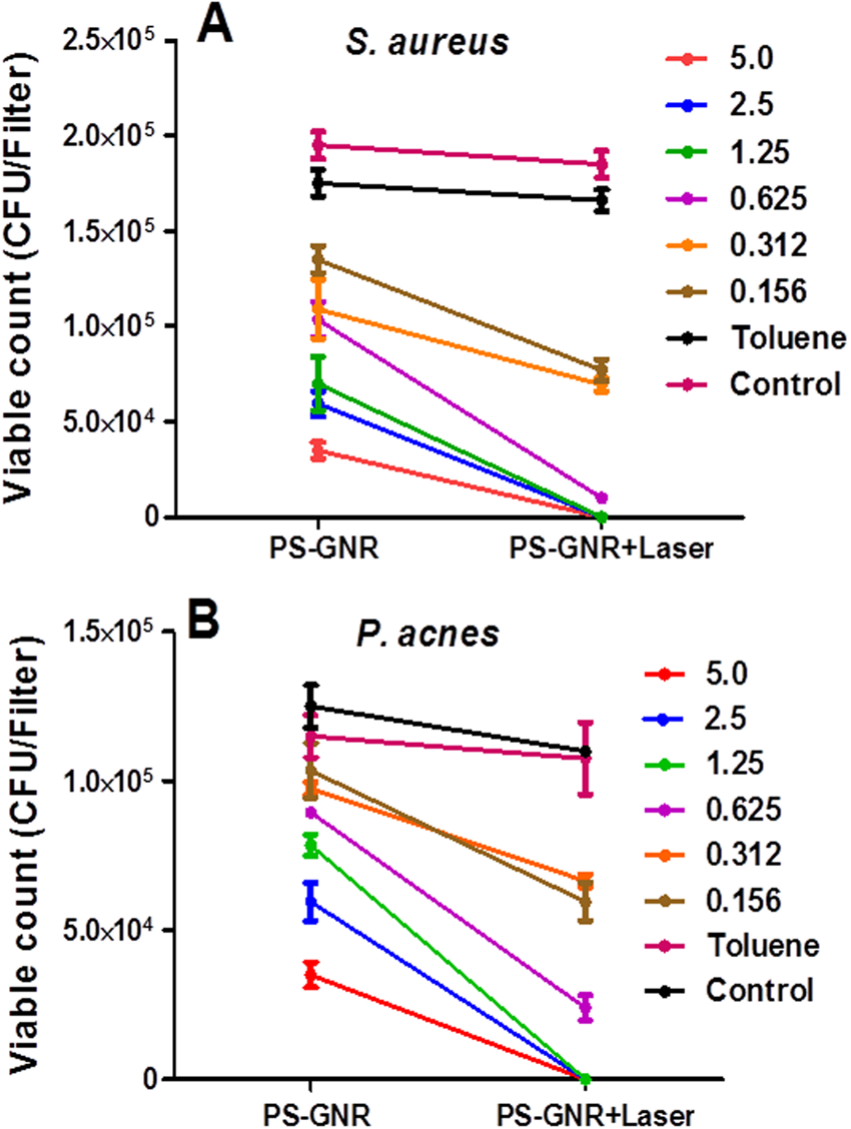
Effect of NIR laser exposure of bacteria pre-treated with PS-GNR. (**A**) Bacterial viable count of *S*. *aureus* treated with different concentrations (nM) of PS-GNR compared to those treated with PS-GNR followed by exposure to laser beam. (**B**) Bacterial viable count of *P*. *acnes* treated with different concentrations (nM) of PS-GNR compared to those treated with PS-GNR followed by exposure to laser beam. Control: bacteria exposed to laser beam alone without GNR.

The bacterial viable counts of *S*. *aureus* and *P*. *acnes* upon exposure to PEG-GNR followed by laser beam exposure were significantly lower than that upon exposure to PEG-GNR alone over the concentration range of 0.25–0.06 nM, Fig. 4A,B. Similarly, the bacterial viable counts of *S*. *aureus* and *P*. *acnes* upon exposure to PS-GNR followed by laser beam exposure were significantly lower than that upon exposure to PS-GNR alone over the concentration range of 5.0–0.625 nM, Fig. 5A,B.

**Figure 6:**
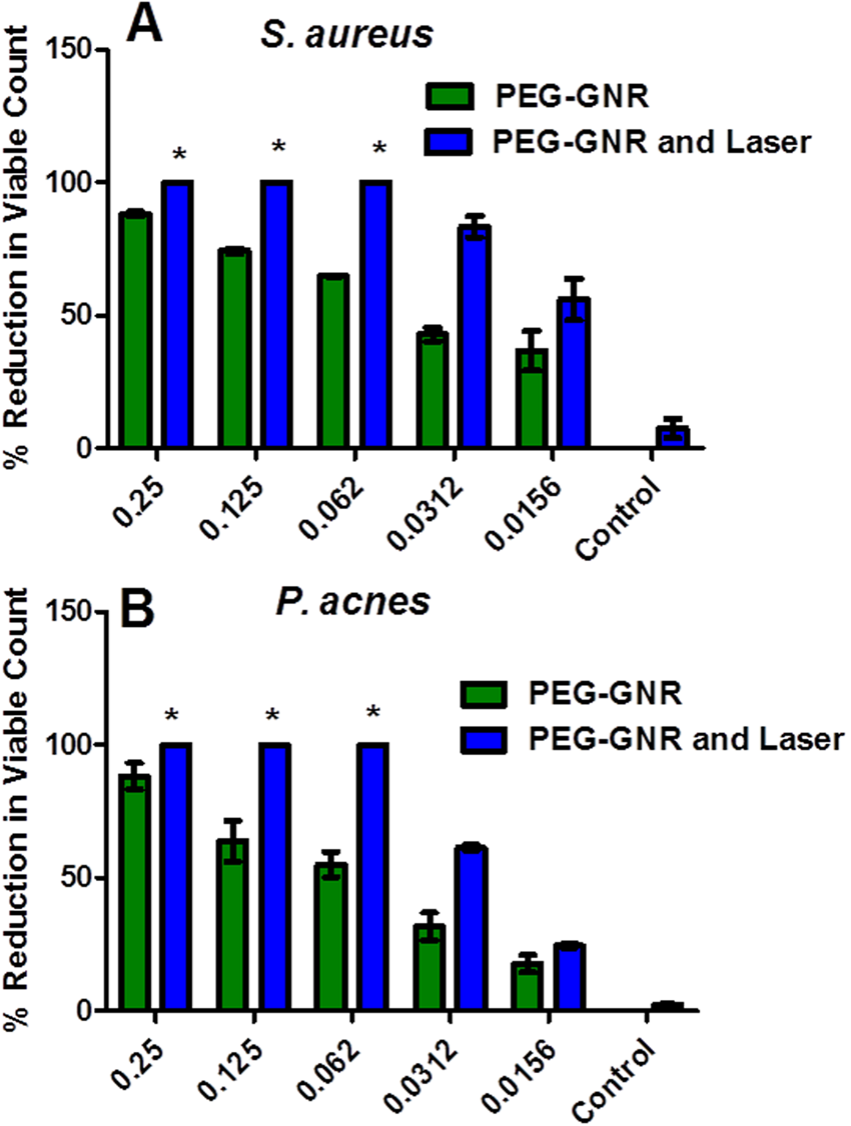
Percentage reduction of bacterial viable count of (**A)** *S*. *aureus* and (**B)** *P*. *acnes* pre-treated with PEG-GNR alone compared to those treated with PEG-GNR followed by laser beam exposure. *Percentage reduction of bacterial viable count ≥99.99%, i.e. ≥4 log_10_ cycles. The standard deviations for the first three bars are very small.

**Figure 7:**
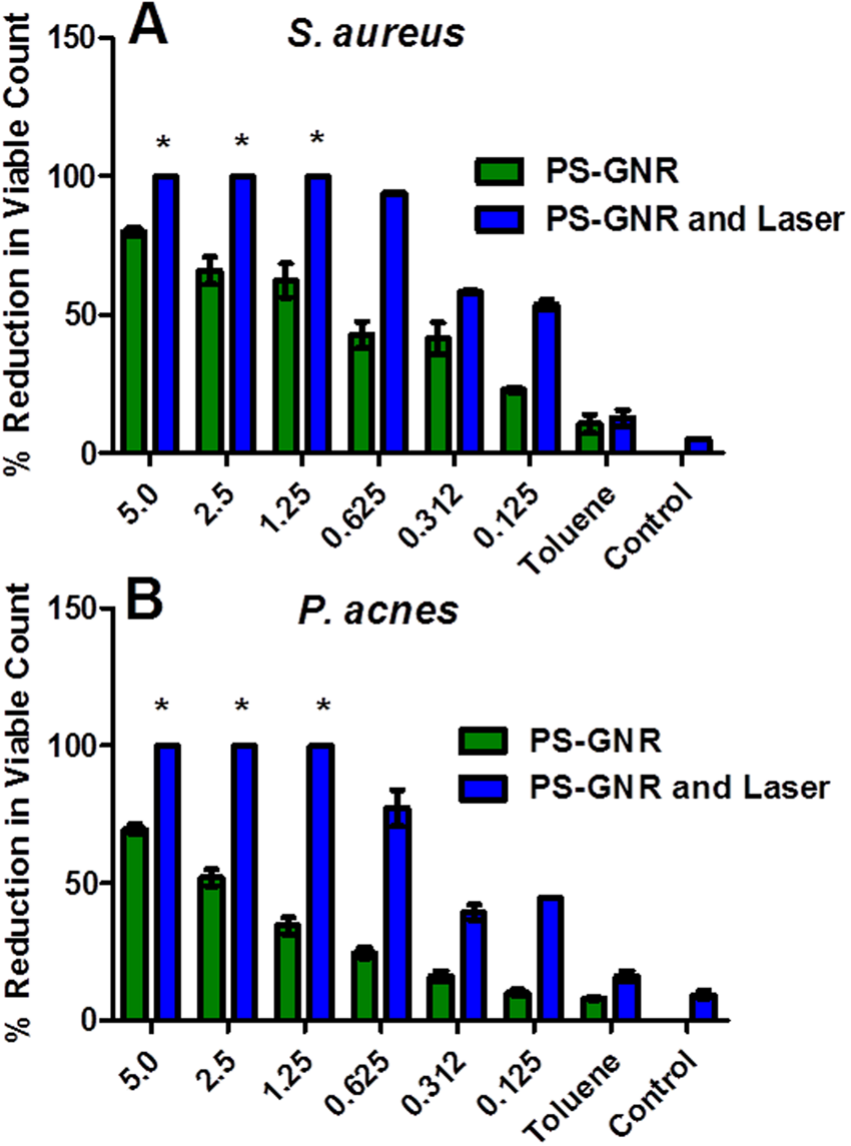
Percentage reduction of bacterial viable count of (**A)** *S*. *aureus* and (**B)** *P*. *acnes* pre-treated with PS-GNR alone compared to those treated with PS-GNR followed by laser beam exposure. *Percentage reduction of bacterial viable count ≥99.99%, i.e. ≥4 log_10_ cycles. The standard deviations for the first three bars are very small.

Interestingly, the exposure to NIR laser (15 min) resulted in ≥99.99%, i.e. ≥4 log_10_ cycles reduction of bacterial viable count of both *S*. *aureus* and *P*. *acnes* that were pre-exposed to PEG-GNR (15 min) at a concentration range of 0.25–0.06 nM compared to those exposed to PEG-GNR alone (≤85%, i.e. ≤1 log_10_ cycle reduction of bacterial viable count) or laser alone (≤10% reduction of bacterial viable count), Fig. 6A,B. Similar killing effect was observed on *S*. *aureus* and *P*. *acnes* that were pre-exposed to PS-GNR at a concentration range of 5.0–1.25 nM compared to those exposed to PS-GNR or laser alone, Fig. 7A,B.

**Figure 8:**
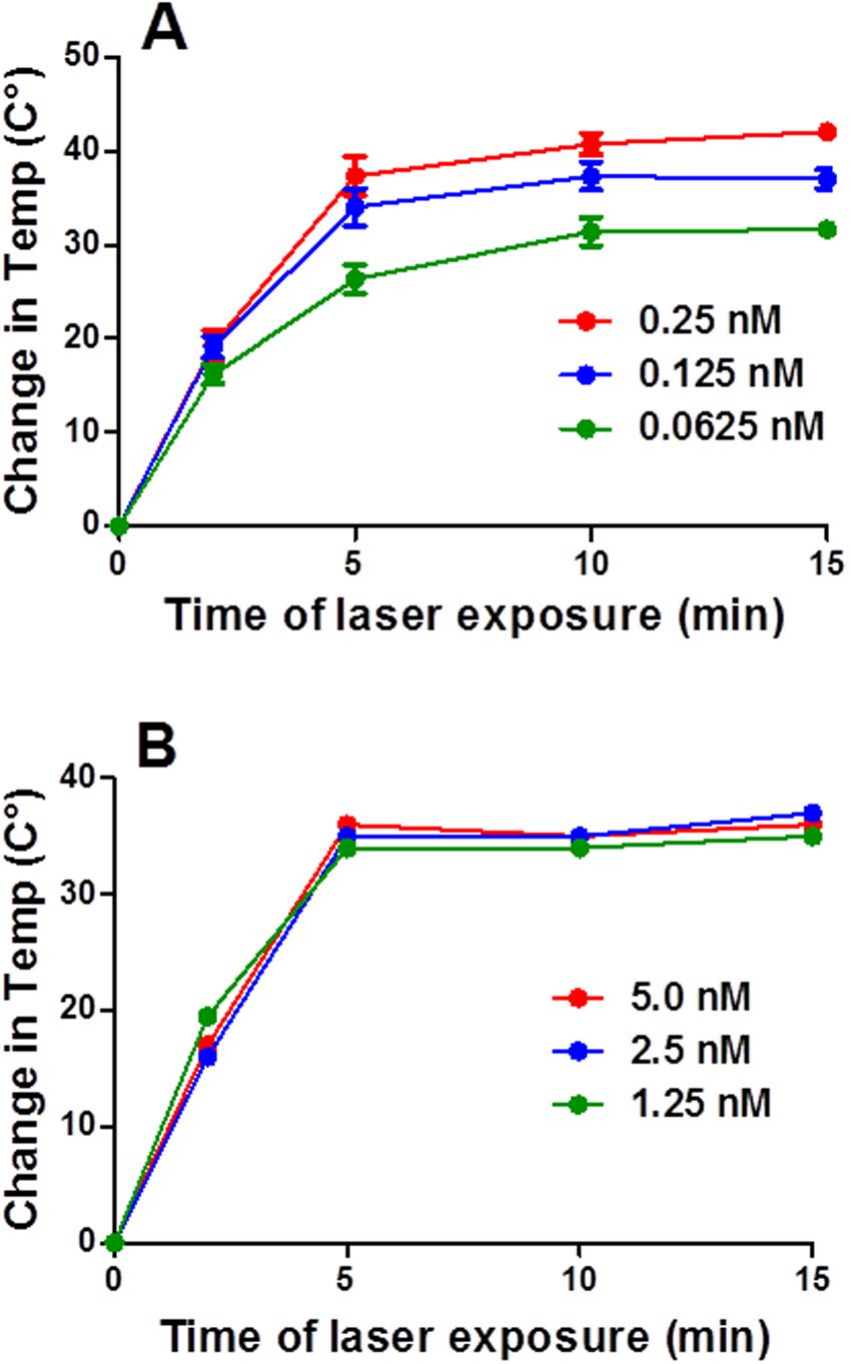
Temperature change induced by photothermal heating of (**A**) PEG-GNR and (**B**) PS-GNR at different molar concentrations and increasing NIR laser exposure times. All initial temperatures were 21 ± 0.5 °C in solutions and 19.2 ± 0.6 °C on agar plates. The standard deviations in (**B**) are very small.

In order to correlate the calculated percentage reduction of bacterial viable count to the photothermal heat conversion, we measured the photothermal heating of the different functionalized GNR at varying concentrations over 15 min (the time of treatment) of NIR laser beam exposure. As shown in Fig. 8A, for PEG-GNR, the temperature increased sharply over the first two minutes (the change of temperature was ~18 °C) regardless of PEG-GNR concentration. The maximum increase in temperature was observed after 10 min of laser exposure across different concentrations of PEG-GNR. However, the highest change in temperature (~43 °C) was observed for the highest concentration. The maximum generated heat upon exposure to NIR laser beam was maintained for the rest 5 min of treatment and it is in well agreement with other reports that investigated the photothermal effect of GNP against bacteria and cancer cells^36,37^. We propose that the light absorbed by NIR-absorbing GNR can be efficiently converted into thermal energy that is responsible for the observed killing effect in addition to the endogenous cytotoxic effect of GNR. On the other hand, the increase of temperature upon exposure of hydrophobic PS-GNR to NIR laser beam was measured directly on the agar plates. Figure 8B shows a gradual rise in temperature over the first 5 min of laser exposure reaching a reported maximum temperature of ~55 °C, which was maintained over the 15 min of laser treatment.

The photothermal conversion efficiency of GNR upon excitation by a laser beam was approved in previous studies, which utilized similar laser type and optical density to that employed in the current study^28,36^. We propose that exposure of PEG-GNR or PS-GNR to the NIR laser resulted in photothermolysis of the tested bacteria even at low concentrations (0.06 nM and 1.25 nM of PEG-GNR and PS-GNR respectively). The photothermal ablation was evidenced by the dramatic thermal elevation of the tested GNR suspensions. However, the bactericidal activity of GNR themselves before laser exposure could not be excluded.

Studies investigated the photothermal ablation activity of functionalized GNR on either *S*. *aureus* or *P*. *acnes* are lacking in the literature. In a recent study, authors demonstrated that the laser-activated GNP treatment (8 ns pulsed laser irradiation at a wavelength of 532 nm and fluences ranging from 1 to 5 J/cm) reduced the bacterial viable count to 31% of control in the methicillin-sensitive *S*. *aureus* population, while the viable count in the MRSA population was reduced to 58% of control^25^. Another study by Castillo-Martínez *et al*. mentioned that the value of minimum bactericidal concentration for CTAB-GNR was much less for *S*. *aureus* exposed to NIR laser (wavelength of 810 nm and a 200 mW broadcast power) compared to that without exposure^38^. Furthermore, Kim *et al*. found that the NIR light provided rapid killing of 99.9% of the *S*. *aureus* and *E*. *coli* exposed to functionalized GNR^37^. However, comparison between the results of the current study and the previous published reports is difficult due to differences in shape and surface functionalities of the tested GNP, in addition to the differences in the type of laser used and its maximum power.

For efficient photothermal therapy, GNP should successfully attach to the bacterial cells, so the localized heating that results during NIR irradiation will cause irreversible and permanent bacterial cellular damage^15,39^. Accordingly, the surface properties of GNR have a determinant role in directing the GNR towards the targeted bacteria in order to maximize their photothermal properties upon laser excitation. Capping the GNR with PEG increases their compatibility with biological media, because it decreases the non-specific protein binding and promotes their stability in the biological matrices, and subsequently improves their uptake by bacteria^40^. Moreover, Xiao-Yan *et al*. demonstrated that the effective absorption of the PEG-GNR by *Staphylococcus* bacteria is a detrimental factor in the photothermal antibacterial activity^31^.

On the other hand, it was reported that cationic and hydrophobic functionalized GNP effectively suppressed the growth of both Gram-negative and Gram-positive bacteria^29^. Accordingly, we suppose that the hydrophobic interactions might be responsible for the antimicrobial activity of PS-GNR on both *S*. *aureus* and *P*. *acnes*. However, the concentration range of PS-GNR needed to achieve the highest percentage of killing is much higher than that for PEG-GNR, which might be attributed to the different methods employed to evaluate the bactericidal activity or might be due to the less bacterial uptake of hydrophobic PS-GNR than PEGylated GNR.

### Characterization of the photothermal antibacterial activity of PEG-GNR on *S*. *aureus* using transmission electron microscope (TEM)

In order to understand the uptake mechanism of PEG-GNR by *S aureus* and the killing effect of NIR laser exposure, TEM images were obtained for untreated *S aureus* (control), *S*. *aureus* exposed to NIR laser beam alone and *S*. *aureus* pre-exposed to PEG-GNR then to laser beam.

**Figure 9:**
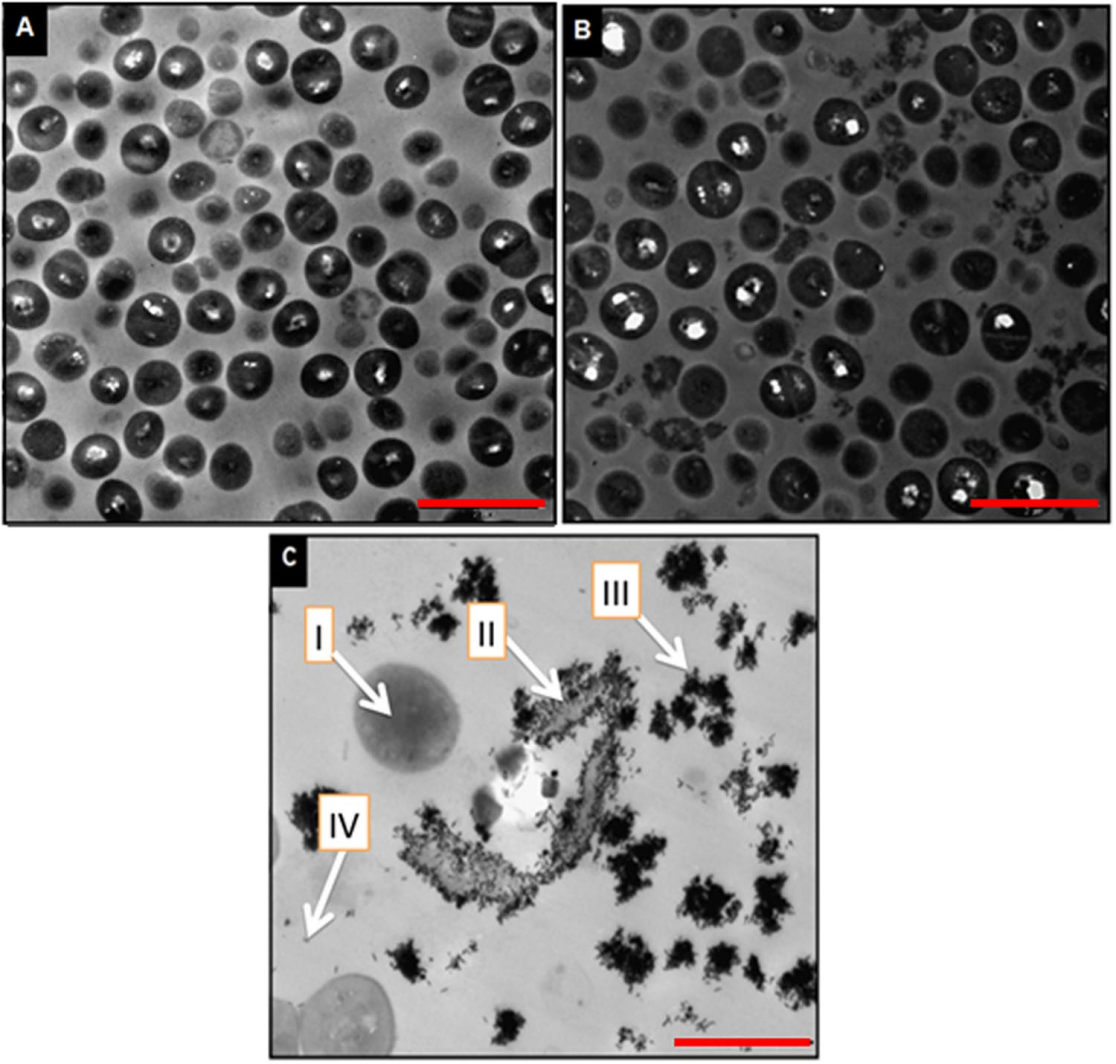
TEM images of (**A)** untreated *S*. *aureus*, (**B**) *S*. *aureus* exposed to laser beam alone and (**C**) *S*. *aureus* pre-treated with PEG-GNR, then exposed to laser beam. (I) A live cell, (II) A dead cell, (III) clusters of GNR, (IV) A single GNR. Scale: A and B = 2 μm, C = 1 μm.

Figure 9A shows TEM image of untreated *S*. *aureus*. The majorities of bacterial cells are intact and have a well-defined cocci shape. Figure 9B shows *S*. *aureus* exposed to NIR laser beam without treatment with PEG-GNR. Although the majorities of bacterial cells are intact and have well defined cocci shape, few cells were disintegrated upon laser exposure. Figure 9C shows TEM image of *S*. *aureus* pre-treated with PEG-GNR, then exposed to NIR laser beam. The image reveals high uptake of PEG-GNR by the cellular wall although we could not exclude the possibility of uptake of PEG-GNR inside cells. The image shows three cells have undergone lysis and disintegration which might be caused by the local heat generated by GNR after exposure to laser irradiation.

Our overall results suggest that the local heat generated upon exposure of functionalized GNR to NIR laser has a significant reduction of bacterial viable count (≥4 log_10_) against skin pathogens such as *S*. *aureus* and *P*. *acnes* compared to functionalized GNR or laser beam alone. Such significant reduction could produce potent antibacterial activity and minimum side effects using minimum concentration of GNR.

## Methods

### Chemicals and instruments

The following materials were obtained from Sigma Aldrich Chemicals, USA: chloroauric acid (HAuCl_4_.3H_2_O, 99.9%), sodium borohydride (NaBH_4_, 99%), silver nitrate (AgNO_3_, 99%), polystyrene thiol (PS-SH), average Mw11,000 g/mole, ascorbic acid (99%), methoxy poly ethylene glycol thiol (m-PEG-SH, Mw ~ 2000 g/mole), cetyltrimethylammonium bromide (CTAB, 99%), poly-L-lysine solution and epoxy embedding medium kit.

Glutaraldehyde (25%) was obtained from Kock-Light Laboratories Ltd., UK. Formvar coated copper TEM grids (300 mesh) were obtained from Ted Pella Inc., Canada. 96-well plates and 24-well plates were obtained from Greiner bio-one, Germany.

Nutrient broth, reinforced clostridial broth, agar bacteriological (agar No. 1), nutrient agar, Mueller Hinton broth, *S*.*aureus* ATCC 29213 and *P*. *acnes* ATCC 11827 were obtained from Oxoid, UK. CO_2_ generator sachets (15%) were obtained from Thermo Fisher Scientific, USA. Cellulose acetate filter membranes (0.20 μm) were obtained from Albet, Germany.

UV-vis absorption spectra of GNR were determined using UV-vis spectrophotometer (Spectrascan80D, Biotech Eng., UK) over the range of 400 to 1100 nm. TEM images were obtained using Versa 3D, FEI, Holland, operating at 30 kV. Zeta potential analysis was performed using Microtrac Zetatrac, Betatek Inc., Canada. Centrifugation of suspensions was performed using Centrifuge Z 216 M, Hermlel, abortechnik, Germany. Laser diode, 808 nm, Max power output ~1250 mW was obtained from Power Technology, Inc., USA. A digital thermometer probe (Extech, FLIR Commercial Systems Inc., USA) was used to measure the temperature of GNR solutions. All chemicals were used as received from suppliers and all solutions used in preparation of GNP were prepared with purified 18.2 MΩ water.

### Synthesis and characterization of CTAB capped GNR (CTAB-GNR), PEG-GNR, and PS-GNR

CTAB-GNR were synthesized and then functionalized with PEG-SH or PS-SH to obtain PEG-GNR, and PS-GNR and were characterized as reported previously^32^.

### Bactericidal activity of PEG-GNR alone or in combination with laser beam on *S*. *aureus* and *P*. *acnes*

Overnight culture of *S*. *aureus* and 72 h anaerobic culture (15% CO_2_) of *P*. *acnes* were used in this study. Percentage reduction of viable count was calculated over different concentrations of GNR using two fold broth micro dilution method in 96-well plates. Double-strength medium (100 μL) of Mueller Hinton broth for *S*. *aureus* or reinforced clostridial broth for *P*. *acnes* was used to fill the first experimental well. The other wells were filled with single-strength medium (100 μL). A volume of 100 μL of PEG-GNR suspensions (4.0 nM) was added to the first well. Two fold serial dilutions were then carried out across the plate. A volume of 10 μL of the cultured microorganisms (*S*. *aureus* or *P*. *acnes*) was used to inoculate each well to achieve an inoculum size of *ca*. 1.5 × 10^6^ CFU/mL. Negative controls were performed with only sterile broth, and positive controls were performed with only bacterial culture with media in the wells. The plates were then incubated aerobically at 37 °C for *S*. *aureus* or anaerobically at 37 °C for *P*. *acnes* for 15 min. After that, wells containing PEG-GNR at concentrations of: 0.25, 0.125, 0.06, 0.03 and 0.015 nM and the positive control were exposed to a laser beam (808 nm, average optical density ~3 W/cm^2^), covered an area of ~0.5 cm^2^ for 15 minutes. A replicate plate was prepared and incubated at 37 °C for 30 min and was not laser treated. Subsequently, bacterial viable count in each well was determined using tenfold serial dilution and the standard spread plate methods, and the percentage of bacterial viable count reduction upon treatment with PEG-GNR alone or after exposure to the laser beam was calculated^41^. From each well, as well as from the control, a volume of 100 μL was transferred to 900 μL of normal saline. Tenfold serial dilutions were carried out and 100 μL of each diluted mixture, were spread onto nutrient agar plates for *S*. *aureus* or reinforced clostridial agar for *P*. *acnes*. Plates were subsequently incubated at 37 °C for 24 hr aerobically for *S*. *aureus* or under anaerobic condition at 37 °C for 72 hr for *P*. *acnes*. Plates contain from 30 to 300 colonies were counted and the viable count of bacteria treated with PEG-GNR alone and those treated with PEG-GNR and laser beam was calculated using Equation (1):

1 $$Test\,count\,result\,(\frac{CFU}{mL})=\frac{No.\,of\,colonies\times Dilution\,factor}{Volume\,of\,culture\,plate\,(mL)}.$$

The percentage of bacterial reduction was calculated using Equation (2):

2 $$ \% \,Reduction=\frac{Initial\,count\,(\frac{CFU}{mL})-Test\,count\,result\,(\frac{CFU}{mL})}{Initial\,count\,(\frac{CFU}{mL})}\times 100.$$

The experiments were done in triplicate.

### Bactericidal activity of PS-GNR alone or in combination with laser beam on *S*. *aureus* and *P*. *acnes*

For determination of antibacterial activity of hydrophobic PS-GNR, the following method was developed and optimized. An equivalent volume of 10 μL of PS-GNR of the following concentrations: 5.0, 2.5, 1.25, 0.625, 0.312, 0.156 nM and toluene (the vehicle used to suspend the PS-GNR) were applied onto the centers of sterile filter membranes (0.2 µm). The filter membranes were then dried and inoculated with a volume of 10 μL of the cultured microorganisms; *S*. *aureus* or *P*. *acnes* at an inoculum size of *ca*. 1.5 × 10^5^ CFU/filter and put onto nutrient agar plates for *S*. *aureus* or onto reinforced clostridial agar plates for *P*. *acnes*. A filter membrane inoculated with bacterial culture (10 μL) was used as a control. The plates were then incubated for 15 min at suitable conditions. After that, filter membranes containing the following concentrations of PS-GNR: 5.0, 2.5, 1.25, 0.625, 0.312, 0.156 and the controls were exposed to a laser beam (808 nm, average optical density ~3 W/cm^2^), covered an area of ~0.5 cm^2^ for 15 minutes. A replicate of inoculated PS-GNR filter paper was prepared and incubated at 37 °C for 30 min without laser treatment.

To assess the bactericidal activity of PS-GNR, the percentage reduction of bacterial viable count was calculated using tenfold serial dilutions and the standard spread plate methods^41^. After incubation, each filter membrane was removed from the plate, soaked into 1000 μL normal saline and vortexed for 1 min. Tenfold serial dilution were carried out and 100 μL of each diluted mixture were spread onto agar plates and processed as described in the previous section. Bacterial viable count and percentage reduction in bacterial viable count were calculated using Equations (1) and (2) respectively.

### Measurements of the photothermal heating of PEG-GNR in solutions and PS-GNR on agar plates upon exposure to laser beam

A 250 μL volume of PEG-GNR (the highest three concentrations) in a 96 well plate was exposed to a NIR laser (808 nm) at an average optical density of ~3 W/cm^2^ and a spot area ~0.5 cm^2^ at increasing exposure times. The changes in temperature of the irradiated PEG-GNR solutions were measured by placing a digital thermometer probe directly into the solutions. The temperature increase of a 250 μL of ultrapure water was also measured upon laser exposure, and the temperature increase of the water was subtracted from that of the PEG-GNR solutions in order to account for any heat generated from the laser itself. All initial temperatures were 21.0 ± 0.5 °C.

The change in temperature of the PS-GNR on the filter membrane upon laser exposure was measured by placing the digital thermometer probe directly on the spot of laser exposure on the agar plate. The temperature of the filter membrane on the agar plate (without PS-GNR) was measured upon laser exposure and the temperature increase of the filter membrane was subtracted from that of the PS-GNR in order to account for any heat generated from the laser itself. All initial temperatures were 19.2 ± 0.6 °C. The experiments were done in triplicate.

### TEM imaging of *S*. *aureus* treated with PEG-GNR and exposed to laser beam

A volume of 500 μL of PEG-GNR (4.0 nM) was mixed with 1.5 mL of cultured *S*. *aureus* (1.5 × 10^6^ CFU/mL) and incubated for 15 min. The mixture was then exposed to the laser beam. Then, the mixture was centrifuged at 12 000 rpm for 12 minutes and the resultant pellets were fixed in 3% glutaraldehyde, post fixed in 2% osmium tetroxide solution and washed with phosphate buffer. The pellets were then dehydrated in ascending series of ethanol and infiltrated in epoxy resin for 24 h. The specimen was polymerized in resin at (60–65 °C). Thin sections (70 nm) were obtained using ultra-microtome and mounted onto a 300 mesh Formvar copper grid for TEM imaging. TEM imaging of bacterial suspension exposed to the laser alone without PEG-GNR was performed as a control.

### Data availability statement

The datasets generated and/or analyzed during the current study are available from the corresponding author on reasonable request.

## References

[1] Owen A (2014). The application of nanotechnology in medicine: treatment and diagnostics. Nanomedicine (Lond)..

[2] Barenholz Y (2012). Doxil®—the first FDA-approved nano-drug: lessons learned. J Control Release..

[3] Gradishar WJ (2005). Phase III trial of nanoparticle albumin-bound paclitaxel compared with polyethylated castor oil-based paclitaxel in women with breast cancer. J Clin Oncol..

[4] Rugo HS (2015). Randomized phase III trial of paclitaxel once per week compared with nanoparticle albumin-bound nab-paclitaxel once per week or Ixabepilone with Bevacizumab as first-line chemotherapy for locally recurrent or metastatic breast cancer: CALGB 40502/NCCTG N063H (Alliance). J Clin Oncol..

[5] Shi J, Kantoff PW, Wooster R, Farokhzad OC (2017). Cancer nanomedicine: progress, challenges and opportunities. Nat. Rev. Cancer..

[6] Ashton S (2016). Aurora kinase inhibitor nanoparticles target tumors with favorable therapeutic index *in vivo*. Sci Transl Med..

[7] Komiyama M, Yoshimoto K, Sisido M, Ariga K (2017). Chemistry can make strict and fuzzy controls for bio-systems: DNA nanoarchitectonics and cell-macromolecular nanoarchitectonics. Bull. Chem. Soc. Jpn..

[8] Her S, Jaffray DA, Allen C (2017). Gold nanoparticles for applications in cancer radiotherapy: mechanisms and recent advancements. Adv. Drug Deliv. Rev..

[9] Choi W, Sahu A, Kim YH, Tae G (2011). Photothermal cancer therapy and imaging based on gold nanorods. Ann. Biomed. Eng..

[10] Alkilany AM, Lohse SE, Murphy CJ (2013). The Gold standard: gold nanoparticle libraries to understand the nano-bio interface. Acc. Chem. Res..

[11] Zhu Y, Ramasamy M, Yi DK (2014). Antibacterial activity of ordered gold nanorodarrays. ACS Appl. Mater. Interfaces..

[12] Olson J (2015). Optical characterization of single plasmonic nanoparticles. Chem Soc Rev..

[13] Kado S, Yokomine S, Kimura K (2017). Widely tunable plasmon resonances from visible to near-infrared of hollow silver nanoshells. Bull. Chem. Soc. Jpn..

[14] Jain PK, Huang XH, El-Sayed IH, El-Sayed MA (2008). Noble metals on the nanoscale: optical and photothermal properties and some applications in imaging, sensing, biology, and medicine. Acc. Chem. Res..

[15] Huang XH, Jain PK, El-Sayed IH, El-Sayed MA (2008). Plasmonic photothermal therapy (PPTT) using gold nanoparticles. Lasers Med. Sci..

[16] Connor EE, Mwamuka J, Gole A, Murphy CJ, Wyatt MD (2005). Gold nanoparticles are taken up by human cells but do not cause acute cytotoxicity. Small..

[17] von Maltzahn G (2009). Computationally guided photothermal tumor therapy using long-circulating gold nanorod antennas. Cancer Res..

[18] Hu M (2006). Gold nanostructures: engineering their plasmonic properties for biomedical applications. Chem Soc Rev..

[19] Zharov VP, Mercer KE, Galitovskaya EN, Smeltzer MS (2006). Photothermal nanotherapeutics and nanodiagnostics for selective killing of bacteria targeted with gold nanoparticles. Biophys J..

[20] Huang X, El-Sayed MA (2010). Gold nanoparticles: Optical properties and implementations in cancer diagnosis and photothermal therapy. J. Adv. Res..

[21] Pustovalov VK, Smetannikov AS, Zharov VP (2008). Photothermal and accompanied phenomena of selective nanophotothermolysis with gold nanoparticles and laser pulses. Laser Phys Lett..

[22] Kim B (2010). Tuning payload delivery in tumor cylindroids using gold nanoparticles. Nat. Nanotech..

[23] Allahverdiyev AM, Abamor ES, Bagirova M, Rafailovich M (2011). Antimicrobial effects of TiO(_2_) and Ag(_2_)O nanoparticles against drug-resistant bacteria and leishmania parasites. Future Microbiol..

[24] Kim CB, Yi DK, Kim PS, Lee W, Kim MJ (2009). Rapid photothermal lysis of the pathogenic bacteria, Escherichia coli using synthesis of gold nanorods. J Nanosci Nanotechnol..

[25] Millenbaugh NJ, Baskin JB, DeSilva MN, Elliott WR, Glickman RD (2015). Photothermal killing of Stapylococcus aureus using antibody-targeted gold nanoparticles. Int J Nanomedicine..

[26] Mocan L (2016). Selective *in vitro* photothermal nano-therapy of MRSA infections mediated by IgG conjugated gold nanoparticles. Sci Rep..

[27] Shokri R, Salouti M, Zanjani RS (2015). Anti protein A antibody-gold nanorods conjugate: a targeting agent for selective killing of methicillin resistant Staphylococcus aureus using photothermal therapy method. J Microbiol..

[28] Zhanga J (2018). Photothermal lysis of pathogenic bacteria by platinum nano dots decorated gold nanorods under near infrared irradiation. J Hazard Mater..

[29] Li X (2014). Functional gold nanoparticles as potent antimicrobial agents against multi-drug-resistant bacteria. ACS Nano..

[30] Khlebtsov BN (2013). Enhanced photoinactivation of Staphylococcus aureus with nanocomposites containing plasmonic particles and hematoporphyrin. J Biophotonics..

[31] Xiao-Yan F, Ying C, Yu-Peng L, Chun-Peng W, Fu-Xiang C (2015). Near-IR photothermal antibacterial effects of polyethylene glycol (peg) modified gold nanorods. Chinese J Inorg Chem..

[32] Mahmoud NN (2017). Preferential accumulation of gold nanorods into human skin hair follicles: Effect of nanoparticle surface chemistry. J Colloid Interface Sci..

[33] Mahmoud NN, Alkilany AM, Khalil EA, Al-Bakri AG (2017). Antibacterial activity of gold nanorods against staphylococcus aureus and propionibacterium acnes: misinterpretations and artifacts. Int J Nanomedicine..

[34] Prabuseenivasan S, Jayakumar M, Ignacimuthu S (2006). *In vitro* antibacterial activity of some plant essential oils. BMC Complement. Altern Med..

[35] Asawahame C (2015). Antibacterial activity and inhibition of adherence of streptococcus mutans by propolis electrospun fibers. AAPS PharmSciTech..

[36] Mackey MA, Ali MRK, Austin LA, Near RD, El-Sayed MA (2014). The most effective gold nanorod size for plasmonic photothermal therapy: theory and *in vitro* experiments. J. Phys. Chem. B..

[37] Kim SH (2015). Light controllable surface coating for effective photothermal killing of bacteria. ACS Appl Mater Interfaces..

[38] Castillo-Martínez, J., Martínez-Castañón, G., Martínez-Gutierrez, F., Zavala-Alonso, N.V. & Patiño-Marín, N. Antibacterial and antibiofilm activities of the photothermal therapy using gold nanorods against seven different bacterial strains. *J Nanomater*. 783671 (2015).

[39] Bucharskaya A (2016). Towards effective photothermal/photodynamic treatment using plasmonic gold nanoparticles. Int J Mol Sci..

[40] Manson J, Kumar D, Meenan B, Dixon D (2011). Polyethylene glycol functionalized gold nanoparticles: the influence of capping density on stability in various media. Gold bulletin..

[41] The Clinical and Laboratories Standards Institutes, M26-A. Methods for determining bactericidal activity of antimicrobial agents. Wayne, PA (1999).

